# A Comprehensive Study on Adverse Reactions of Benzoyl Peroxide (BPO) in Dermatological Aesthetics Utilizing the FAERS Database

**DOI:** 10.1111/jocd.16787

**Published:** 2025-01-14

**Authors:** Siyuan Zhou, Su Yan, Shaopeng Ming

**Affiliations:** ^1^ The Second Affiliated Hospital of Guangxi Medical University Nanning China

**Keywords:** adverse drug events, benzoyl peroxide, carcinogenic, EPSOLAY, FAERS

## Abstract

**Background:**

Benzoyl peroxide (BPO) is widely used in dermatological aesthetics for treating acne and other skin conditions. However, its potential adverse reactions remain a concern.

**Aims:**

This study aimed to investigate the application of BPO in dermatological aesthetics, analyze its associated adverse reactions, and provide insights into patient safety.

**Methods:**

The study analyzed adverse reaction reports related to BPO in the Food and Drug Administration's Adverse Event Reporting System database from 2004 to 2024. Demographic characteristics, types of adverse reactions, and specific issues related to the novel drug EPSOLAY (5% microencapsulated BPO) were examined.

**Results:**

The findings reveal that BPO users are predominantly female (74.18%), with a significant proportion falling within the 18–44 age group (37.45%). The most common adverse reactions involve skin and subcutaneous tissue disorders (36.34%), including skin swelling, pain, and burning sensations. Reports on EPSOLAY indicate adverse reactions such as erythema, pruritus, and skin exfoliation, but no tumor‐related reports have been recorded.

**Conclusions:**

BPO usage in dermatological aesthetics is associated with various adverse reactions, primarily affecting the skin. The introduction of EPSOLAY has not altered the adverse reaction profile significantly. However, the risk of BPO decomposing into the carcinogen benzene under certain conditions necessitates enhanced patient education, improved production processes, and continuous post‐marketing surveillance to ensure drug safety.

## 1 Introduction

Benzoyl Peroxide (BPO), a chemical widely utilized in dermatological aesthetics, particularly in acne treatments, has garnered significant attention because of its potential adverse effects amidst its proven efficacy. BPO has gained widespread acceptance for its effectiveness in combating acne [1]. Through its potent oxidizing properties, BPO eliminates *Propionibacterium acnes* , the bacterium associated with acne inflammation, and exerts anti‐inflammatory, keratolytic, and comedolytic effects, thereby improving skin conditions. Consequently, BPO is a staple in various acne treatment products, favored by many consumers seeking to address acne concerns. In particular, the newly approved 5% microencapsulated BPO (EPSOLAY) in April 2024 encapsulates 5% BPO in silica microcapsules. This technology enables the drug to be slowly released to the skin surface during application, thereby prolonging the therapeutic effect and significantly reducing the potential risk of skin irritation [2].

Despite BPO's effectiveness, its potential adverse effects cannot be overlooked. As a strong oxidant, BPO can irritate the skin, leading to contact dermatitis, pruritus, erythema, dryness, and desquamation. These reactions not only compromise the user experience but may also exacerbate skin problems. Furthermore, recent research suggests that BPO may decompose under high temperatures or specific conditions, releasing harmful substances such as benzene, a known genotoxic carcinogen [3]. Long‐term or excessive exposure to benzene may increase the risk of developing malignancies like leukemia [4], raising significant concerns given the prevalence of BPO in acne treatment products and the multitude of factors that may influence its decomposition during use [5].

The Food and Drug Administration's Adverse Event Reporting System (FAERS), an authoritative drug safety database, is a valuable resource for monitoring and assessing drug safety after market approval. With comprehensive coverage and extensive real‐time information on adverse drug events (ADEs), FAERS is a vital tool for drug vigilance and risk assessment [6].

This study aimed to comprehensively review the current status of BPO application in dermatological aesthetics and its potential adverse reactions. By systematically analyzing the reports on BPO‐related adverse events in the FAERS database, the study seeks to explore the mechanisms, influencing factors, and preventive measures of BPO‐induced adverse reactions. The significance of this research lies in providing scientific reference for clinicians and consumers to guide the rational use of acne‐treating products containing BPO. Additionally, it offers robust support for relevant regulatory bodies to formulate more scientific and reasonable regulatory policies.

## 2 Methods

### 2.1 Data Acquisition

Given the market entry of BPO in 2004, we retrieved relevant data from the FAERS database, covering the period from the first quarter of 2004 to the second quarter of 2024. This dataset encompassed multiple tables, including demographics and administrative information (DEMO), drug information (DRUG), adverse reaction events (REAC), patient outcomes (OUTC), reporting sources (RPSR), therapy start and end dates (THER), indications (INDI), and deleted cases.

**FIGURE 1:**
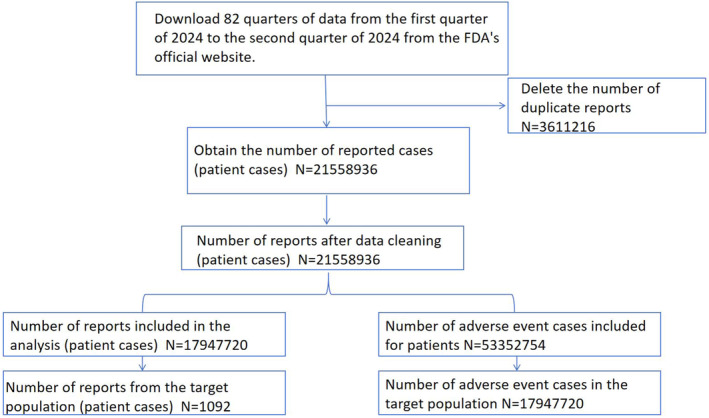
Data acquisition flowchart.

After importing the data into MySQL 15.0, we applied rigorous filtering criteria to isolate records specifically related to BPO. Additionally, we ensured data integrity by removing any duplicate cases to prevent bias in our analysis. The data extraction process is shown in Figure 1.

### 2.2 Analytical Method

First, we will re‐encode ADE reports using a specific dictionary or terminology set in order to standardize the diverse and complex descriptions of advers drug reaction (ADR), facilitating statistical research. This study utilizes the System Organ Class (SOC) and Preferred Term (PT) in the Medical Dictionary for Regulatory Activities (MedDRA 25.0) [7] for the standardized delineation and classification of ADEs, with SOC and PT as the research objects.

### 2.3 Calculate ADR Signal

In this study, the reporting odds ratio (ROR) method is employed to calculate ADR signals using the disproportionality approach. The ROR significantly stands out for its ability to synthesize results from multiple studies on drug adverse reactions while accounting for heterogeneity among studies, such as differences in design, sample characteristics, and adverse reaction definitions. This comprehensive analytical approach provides robust and accurate estimates, avoiding biases associated with simple aggregation, particularly in studies with small sample sizes or limited statistical power. The ROR is capable of revealing consistent patterns of adverse reactions, offering crucial references for drug development and clinical practice [8].

In the ROR method, the detection of a signal indicating a potential association between a drug and a specific adverse event occurs when the calculated result exceeds a predefined threshold. Specifically, the ROR is computed using the formula:

$$\mathrm{ROR}=\left(a/b\right)/\left(c/d\right)$$

where *a* represents the number of reports of the target adverse event for the target drug, *b* represents the number of reports of other adverse events for the target drug, *c* represents the number of reports of the target adverse event for nontarget drugs, and *d* represents the number of reports of other adverse events for nontarget drugs.

In the ROR method, a threshold is typically set (ROR ≥ 2). When the ROR value exceeds this threshold, it is considered a signal. Additionally, it is important to consider the confidence interval of the ROR, typically requiring the lower limit of the 95% confidence interval to be greater than 1, to enhance the reliability of the signal [9]. In this study, we utilized Microsoft Excel 2016 to perform calculations related to the ROR.

Lastly, we extracted detailed information from patients who experienced severe adverse reactions, as well as the adverse reactions of the new drug EPSOLAY, to analyze the causes of severe adverse reactions and make an initial assessment of the safety of EPSOLAY.

## 3 Results

### 3.1 Demographic Characteristics of Adverse Reactions to BPO

**FIGURE 2:**
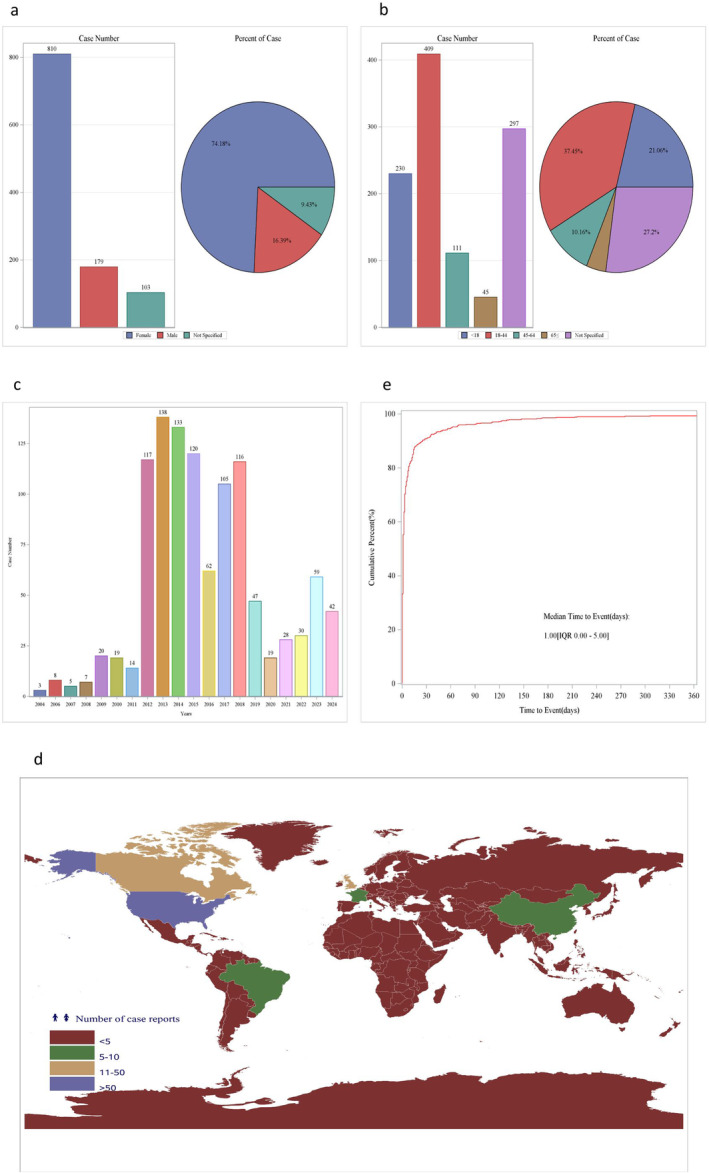
Demographic characteristics. (a) Gender distribution, (b) Age distribution, (c) Time distribution, (d) Regional distribution, (e) Time–adverse reaction rate curve.

Through screening and matching, a total of 1092 patients with 3594 adverse reaction reports were analyzed for demographic characteristics. The gender distribution showed a predominance of female patients, accounting for 74.18% (810 cases) of the total reported cases, whereas male patients comprised 16.39% (179 cases). Gender was not specified for 9.43% (103 cases) of the reports (Figure 2a).

Regarding age, patients in the 18–44 age group had the highest percentage of adverse reactions, contributing 37.45% (409 cases) to the total. Patients younger than 18 years and those aged 45–64 years followed, with 21.06% (230 cases) and 10.16% (111 cases) respectively. Only 4.12% (45 cases) of reports were from patients aged 65 years and above. Notably, 27.20% (297 cases) of the reports did not specify the age of the patients (Figure 2b).

Over the reporting years from 2004 to 2024, a gradual increase in the number of adverse reaction reports was observed, with the highest percentage of reports in 2013 (12.64%, 138 cases) and 2014 (12.18%, 133 cases). The years 2004 and 2006 had the lowest number of reports, accounting for 0.27% (3 cases) and 0.73% (8 cases) respectively (Figure 2c).

Most reports (61.90%, 676 cases) were submitted by consumers, followed by reports from other health professionals (5.04%, 55 cases), pharmacists (3.02%, 33 cases), physicians (2.38%, 26 cases), and lawyers (0.37%, 4 cases). A significant proportion of 27.29% (298 cases) of reports did not specify the reporter's occupation.

Geographically, the United States (40.75%, 445 cases) reported the highest number of adverse reactions to BPO, followed by Not Specified (54.21%, 592 cases), the United Kingdom (1.01%, 11 cases), and China (0.73%, 8 cases). Other countries reported fewer cases, with most contributing less than 1% to the total (Figure 2d).

Regarding the administration route, topical administration accounted for the majority of adverse reactions (55.77%, 609 cases), whereas the administration route was not specified for 40.11% (438 cases) of the reports. Intradermal, transdermal, oral, cutaneous, subdermal, dental, respiratory (inhalation), subcutaneous, and transplacental routes collectively accounted for a minor percentage of the total reported cases.

The most common adaptation disease reported was acne, accounting for 75.18% (821 cases) of the total adverse reactions. Product used for unknown indication was reported in 7.88% (86 cases) of the cases, whereas rosacea, therapeutic skin care topical, removal of inert matter from skin or subcutaneous tissue, skin disorder, skin hyperpigmentation, hidradenitis, rash, and other rare indications each contributed less than 2% of the total reports.

In terms of seriousness, 81.59% (891 cases) of the reports were classified as serious, whereas 18.41% (201 cases) were non‐serious. Half of the adverse reactions occurred within one day of medication administration (Figure 2e). Detailed demographic characteristics are presented in Table 1.

**TABLE 1:** Demographic information on adverse reactions to BPO.

Index	Reporting cases (%)
Gender	
Female (%)	810 (74.18)
Male (%)	179 (16.39)
Not specified (%)	103 (9.43)
Age	
< 18 (%)	230 (21.06)
18–44 (%)	409 (37.45)
45–64 (%)	111 (10.16)
65 ≤ (%)	45 (4.12)
Not specified (%)	297 (27.20)
Reporting year	
2004 (%)	3 (0.27)
2006 (%)	8 (0.73)
2007 (%)	5 (0.46)
2008 (%)	7 (0.64)
2009 (%)	20 (1.83)
2010 (%)	19 (1.74)
2011 (%)	14 (1.28)
2012 (%)	117 (10.71)
2013 (%)	138 (12.64)
2014 (%)	133 (12.18)
2015 (%)	120 (10.99)
2016 (%)	62 (5.68)
2017 (%)	105 (9.62)
2018 (%)	116 (10.62)
2019 (%)	47 (4.30)
2020 (%)	19 (1.74)
2021 (%)	28 (2.56)
2022 (%)	30 (2.75)
2023 (%)	59 (5.40)
2024 (%)	42 (3.85)
Reporter	
Consumer (%)	676 (61.90)
Lawyer (%)	4 (0.37)
Not specified (%)	298 (27.29)
Other health professional (%)	55 (5.04)
Pharmacist (%)	33 (3.02)
Physician (%)	26 (2.38)
Occurrence country	
Not specified (%)	592 (54.21)
United States of America (%)	445 (40.75)
United Kingdom (%)	11 (1.01)
China (%)	8 (0.73)
France (%)	5 (0.46)
Canada (%)	5 (0.46)
Russia (%)	3 (0.27)
Italy (%)	3 (0.27)
Brazil (%)	2 (0.18)
Belgium (%)	2 (0.18)
Portugal (%)	2 (0.18)
Japan (%)	2 (0.18)
Sweden (%)	2 (0.18)
Ireland (%)	1 (0.09)
Austria (%)	1 (0.09)
Australia (%)	1 (0.09)
Puerto Rico (%)	1 (0.09)
Germany (%)	1 (0.09)
Finland (%)	1 (0.09)
South Africa (%)	1 (0.09)
Norway (%)	1 (0.09)
Uganda (%)	1 (0.09)
Chile (%)	1 (0.09)
Administration route	
Topical (%)	609 (55.77)
Not specified (%)	438 (40.11)
Intradermal (%)	16 (1.47)
Transdermal (%)	8 (0.73)
Unknown (%)	7 (0.64)
Oral (%)	4 (0.37)
Cutaneous (%)	3 (0.27)
Subdermal (%)	3 (0.27)
Dental (%)	1 (0.09)
Respiratory (inhalation) (%)	1 (0.09)
Subcutaneous (%)	1 (0.09)
Transplacental (%)	1 (0.09)
Adaptation disease	
Acne (%)	821 (75.18)
Not specified (%)	144 (13.19)
Product used for unknown indication (%)	86 (7.88)
Rosacea (%)	13 (1.19)
Therapeutic skin care topical (%)	4 (0.37)
Removal of inert matter from skin or subcutaneous tissue (%)	3 (0.27)
Skin disorder (%)	3 (0.27)
Skin hyperpigmentation (%)	3 (0.27)
Hidradenitis (%)	2 (0.18)
Rash (%)	2 (0.18)
Acne cystic (%)	1 (0.09)
Acne, prophylaxis (%)	1 (0.09)
Acne, skin disorder (%)	1 (0.09)
Erectile dysfunction (%)	1 (0.09)
Hypersensitivity (%)	1 (0.09)
Occupational exposure to product (%)	1 (0.09)
Oil acne (%)	1 (0.09)
Pain (%)	1 (0.09)
Product use in unapproved indication (%)	1 (0.09)
Rash papular (%)	1 (0.09)
Suicide attempt (%)	1 (0.09)
Serious report	
Serious (%)	891 (81.59)
Non‐serious (%)	201 (18.41)
Time of adverse event—date of medication (days)	
*N* (missing)	718 (374)
Mean (SD)	26.19 (353.04)
Median (Q1, Q3)	1.00 (0.00, 5.00)
Min, max	0.00, 9374.00

### 3.2 Results of Adverse Reaction Analysis

**FIGURE 3:**
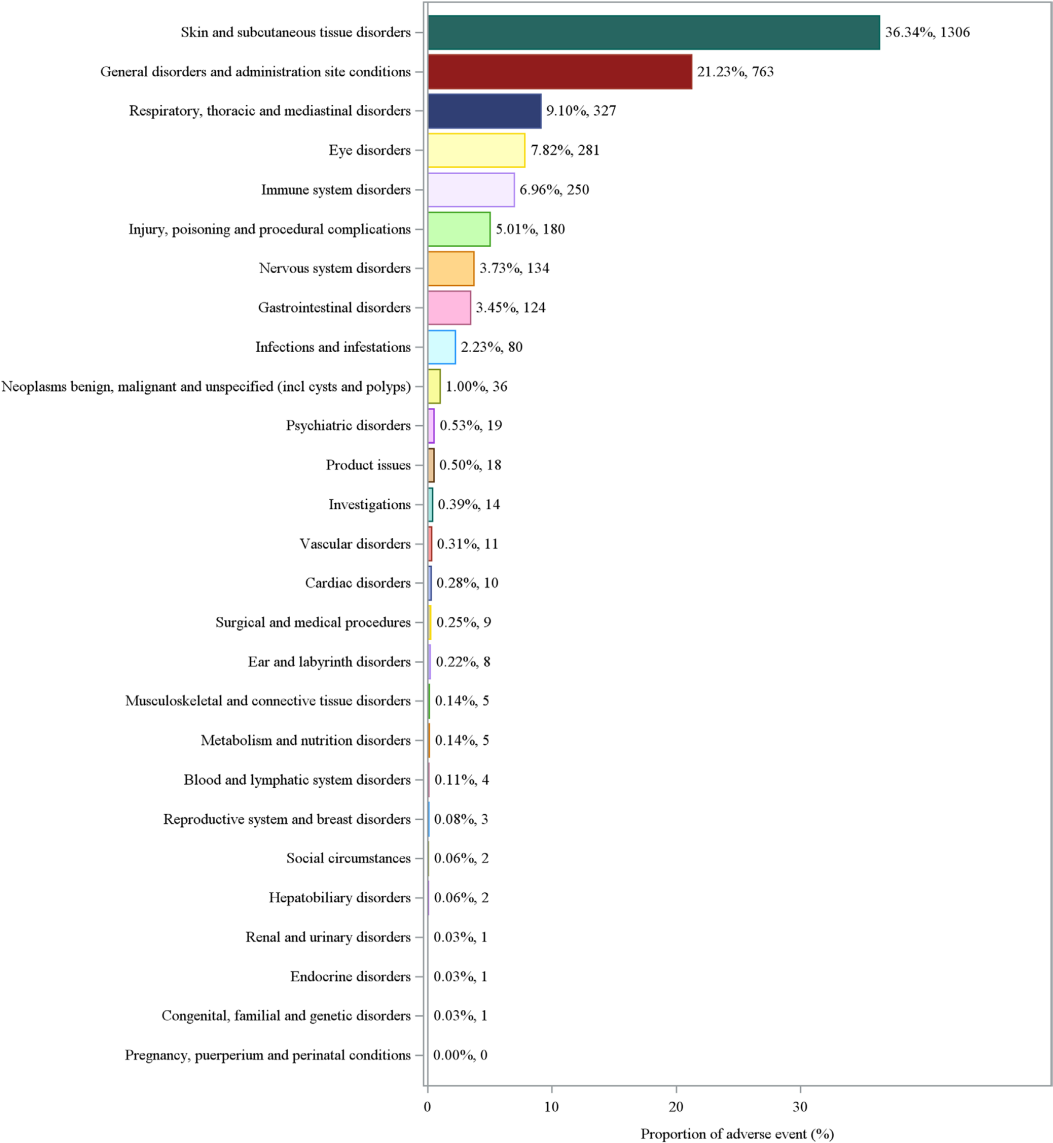
Proportion of adverse events by SOC.

**FIGURE 4:**
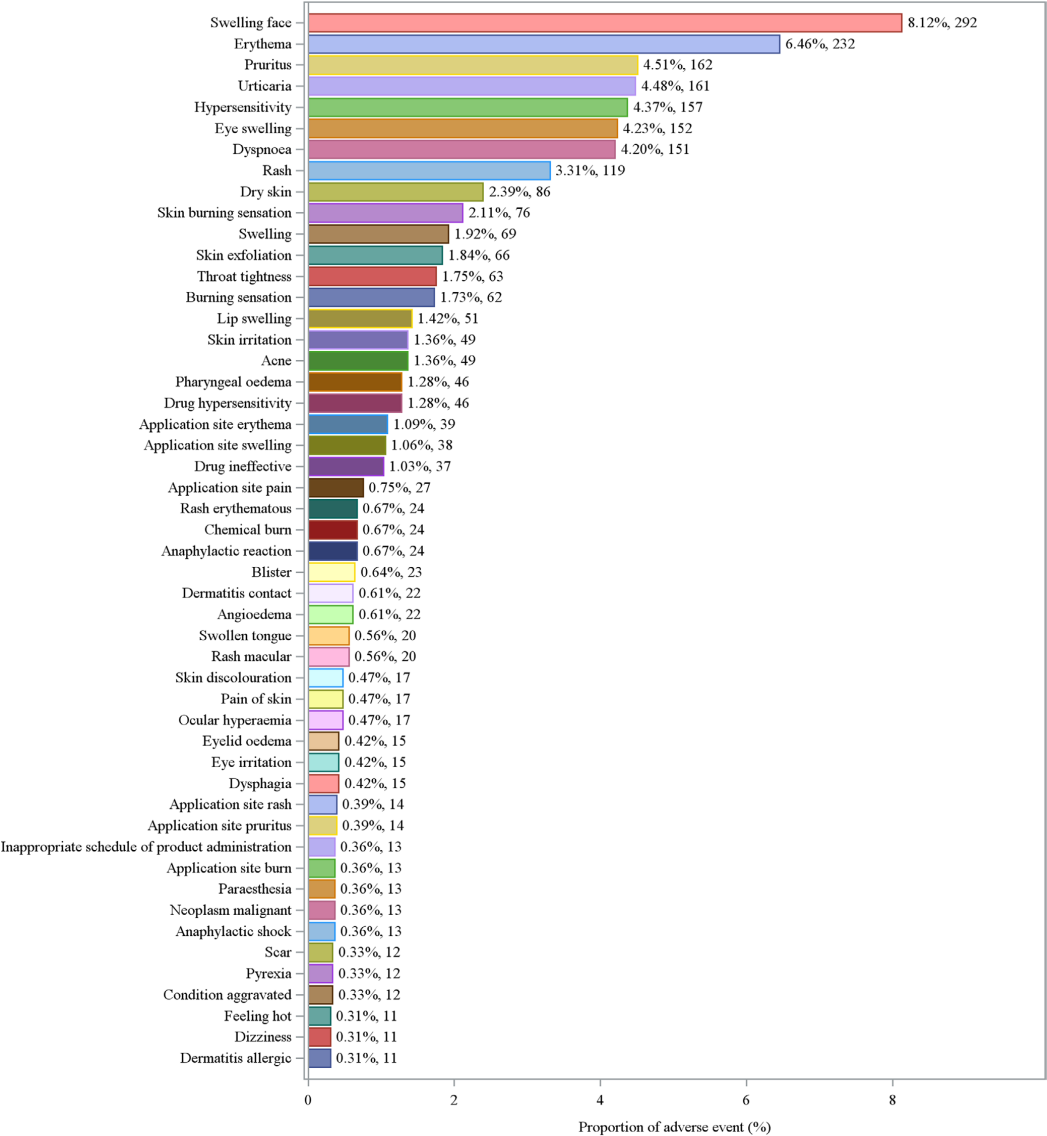
Proportion of adverse events by PT.

The adverse reaction distribution according to SOC indicates that skin and subcutaneous tissue disorders account for the highest proportion (36.34%), followed by general disorders and administration site conditions (21.23%). Respiratory, thoracic, and mediastinal disorders, eye disorders, and immune system disorders also occupy a certain percentage, whereas other SOC categories, such as psychiatric disorders and blood and lymphatic system disorders, contribute to a lower proportion (Figure 3). On the basis of the PT analysis results, it can be observed that the adverse reactions reported for BPO are predominantly skin‐related, including common symptoms such as swelling, pain, burning sensation, and rash (Figure 4).

By calculating the ROR values, we have identified a total of five types of organ‐related adverse reaction signals. Among these, the strongest adverse reaction signal pertains to “Skin and subcutaneous tissue disorders.” The detailed results are presented in Table 2.

**TABLE 2:** Adverse reaction signals classified by SOC (sorted by ROR value and ROR value 95% CI lower).

SOC	Events	ROR值	ROR (95% CI lower)	ROR (95% CI upper)
Skin and subcutaneous tissue disorders	1306	10.04	9.38	10.75
Immune system disorders	250	6.74	5.93	7.66
Eye disorders	281	4.20	3.72	4.74
Respiratory, thoracic, and mediastinal disorders	327	2.03	1.81	2.27
General disorders and administration site conditions	763	1.28	1.18	1.38

On the basis of the PT analysis results, it can be observed that the adverse reactions reported for BPO are predominantly skin related, including common symptoms such as swelling, pain, burning sensation, and rash. The top 30 adverse reaction signals, classified by PT, are detailed in Table 3.

**TABLE 3:** Top 30 adverse reaction signals classified by PT (sorted by ROR value and ROR value 95% CI lower).

PT	PT code	Events	ROR value	ROR (95% CI lower)	ROR (95% CI upper)
Chemical burn	10008420	24	174.52	116.55	261.33
Application site swelling	10053424	38	124.52	90.33	171.64
Swelling face	10042682	292	83.59	74.14	94.24
Eye swelling	10015967	152	74.68	63.46	87.88
Pharyngeal oedema	10034829	46	45.77	34.21	61.25
Throat tightness	10043528	63	40.55	31.60	52.04
Dermatitis contact	10012442	22	34.27	22.52	52.13
Lip swelling	10024570	51	26.55	20.14	35.02
Application site erythema	10003041	39	24.00	17.50	32.91
Application site pain	10003051	27	20.22	13.85	29.54
Erythema	10015150	232	19.98	17.49	22.82
Skin burning sensation	10054786	76	18.40	14.66	23.10
Urticaria	10046735	161	17.93	15.30	21.00
Skin irritation	10040880	49	17.87	13.47	23.69
Hypersensitivity	10020751	157	15.24	12.98	17.88
Burning sensation	10006784	62	15.15	11.78	19.47
Skin exfoliation	10040844	66	14.38	11.27	18.35
Dry skin	10013786	86	12.14	9.80	15.03
Swelling	10042674	69	10.92	8.61	13.86
Acne	10000496	49	10.64	8.03	14.11
Rash macular	10037867	20	10.19	6.57	15.82
Rash erythematous	10037855	24	9.74	6.52	14.55
Angioedema	10002424	22	8.07	5.31	12.27
Anaphylactic reaction	10002198	24	7.96	5.33	11.90
Pruritus	10037087	162	7.79	6.65	9.12
Blister	10005191	23	7.34	4.87	11.07
Dyspnoea	10013968	151	4.72	4.01	5.55
Rash	10037844	119	4.68	3.90	5.62
Drug hypersensitivity	10013700	46	4.01	3.00	5.36

From the analysis of tumor‐related adverse reactions, we have observed that a considerable portion of them have developed into adverse reaction signals, primarily associated with two products: Proactive+ Skin Smoothing Exfoliator (a skin smoothing exfoliator) and Proactive Solution Renewing Cleanser (a renewing cleanser). Both products are topical medications primarily used for acne treatment. Most of these adverse reactions were reported by consumers, and all were administered topically, with formulations including cream and gel (Table 4).

**TABLE 4:** Details of tumor‐related adverse reactions.

Case ID	PT	Drug indication	Drug name	Administration route	Dose form	Reporter	ROR signal (Y=Yes, N=No)
22725637	Neoplasm malignant	Not specified	Proactiv+ Skin Smoothing Exfoliator	Topical	Cream	Consumer	Y
22815081	Neoplasm malignant	Not specified	Proactiv+ Skin Smoothing Exfoliator	Topical	Cream	Consumer	Y
22897750	Neoplasm malignant	Not specified	Proactiv Solution Renewing Cleanser	Topical	Gel	Consumer	Y
22926449	Neoplasm malignant	Not specified	Proactiv Solution Renewing Cleanser	Topical	Gel	Consumer	Y
23244856	Neoplasm malignant	Not specified	Proactiv Solution Renewing Cleanser	Topical	Not specified	Consumer	Y
23292395	Neoplasm malignant	Not specified	Proactiv+ Skin Smoothing Exfoliator	Topical	Not specified	Consumer	Y
23296384	Neoplasm malignant	Not specified	Proactiv Solution Renewing Cleanser	Topical	Gel	Consumer	Y
23304927	Neoplasm malignant	Not specified	Proactiv+ Skin Smoothing Exfoliator	Topical	Not specified	Consumer	Y
23346331	Neoplasm malignant	Not specified	Proactiv Solution Renewing Cleanser	Topical	Gel	Consumer	Y
23512617	Neoplasm malignant	Not specified	Proactiv Solution Renewing Cleanser	Topical	Not specified	Not specified	Y
23545374	Neoplasm malignant	Not specified	Proactiv Solution Renewing Cleanser	Topical	Not specified	Not specified	Y
23548924	Neoplasm malignant	Not specified	Proactiv+ Skin Smoothing Exfoliator	Topical	Not specified	Not specified	Y
23613604	Neoplasm malignant	Not specified	Proactiv Solution Renewing Cleanser	Topical	Not specified	Not specified	Y
11374101	Skin cancer	Acne	Proactiv+ Skin Smoothing Exfoliator	Not specified	Not specified	Consumer	Y
23115359	Skin cancer	Acne	Proactiv+ Skin Smoothing Exfoliator	Topical	Not specified	Consumer	Y
23155087	Skin cancer	Not specified	Proactiv Solution Renewing Cleanser	Topical	Not specified	Consumer	Y
23398108	Skin cancer	Not specified	Proactiv Solution Renewing Cleanser	Topical	Not specified	Not specified	Y
23527767	Skin cancer	Not specified	Proactiv Solution Renewing Cleanser	Topical	Not specified	Not specified	Y
23527770	Skin cancer	Not specified	Proactiv Solution Renewing Cleanser	Topical	Not specified	Not specified	Y
23544386	Skin cancer	Not specified	Proactiv Solution Renewing Cleanser	Topical	Not specified	Not specified	Y
23938696	Skin cancer	Not specified	Proactiv Solution Renewing Cleanser	Topical	Not specified	Not specified	Y
17577630	Breast cancer	Acne	Proactiv Solution Renewing Cleanser	Topical	Cutaneous solution	Consumer	N
23331771	Breast cancer	Not specified	Proactiv Solution Renewing Cleanser	Topical	Not specified	Consumer	N
23613606	Breast cancer	Not specified	Proactiv Solution Renewing Cleanser	Topical	Not specified	Not specified	N
23819694	Breast cancer	Not specified	Proactiv Solution Renewing Cleanser	Topical	Not specified	Not specified	N
23977137	Acute lymphocytic leukemia	Not specified	Proactiv Solution Renewing Cleanser	Topical	Not specified	Consumer	N
23588717	Breast cancer stage III	Not specified	Proactiv Solution Renewing Cleanser	Topical	Not specified	Consumer	N
23647386	Endometrial cancer metastatic	Not specified	Proactiv Solution Renewing Cleanser	Topical	Not specified	Consumer	N
12598712	Leukemia	Acne (痤疮)	Proactiv+ Skin Smoothing Exfoliator	Topical		Consumer	N
23378294	Lung adenocarcinoma stage III	Not specified	Proactiv+ Skin Smoothing Exfoliator	Topical		Not specified	N
10384118	Pancreatic carcinoma	Acne (痤疮)	Proactiv+ Skin Smoothing Exfoliator	Not specified		Not specified	N
23358579	Plasma cell myeloma	Not specified	Proactiv Solution Renewing Cleanser	Topical		Not specified	N
23797864	Renal cancer stage IV	Not specified	Proactiv Solution Renewing Cleanser	Topical		Not specified	N
23819694	Gastrointestinal cancer metastatic	Not specified	Proactiv Solution Renewing Cleanser	Topical		Not specified	N
22725637	Brain neoplasm	Not specified	Proactiv+ Skin Smoothing Exfoliator	Topical	Cream	Consumer	N
17577630	Colorectal cancer	Acne (痤疮)	Proactiv Solution Renewing Cleanser	Topical	Cutaneous solution	Consumer	N

Although EPSOLAY has been introduced to the market for a relatively short period, several adverse reaction events have been reported. We have extracted patient information pertaining to adverse reactions associated with EPSOLAY and have identified several key points and trends. These adverse reactions encompass a diverse range of dermatological symptoms, including erythema, pruritus, skin exfoliation, skin swelling, and skin burning sensation, with the majority occurring shortly after patients commenced EPSOLAY use (some even occurring on the same day of medication). Furthermore, the reporters of these adverse reactions encompass consumers, physicians, and pharmacists, reflecting feedback from various groups after EPSOLAY usage. Notably, some patients also reported issues such as drug inefficacy, improper medication administration (including inadequate dosage, expired products, and inappropriate medication regimens), as well as unapproved indication usage (Table 5).

**TABLE 5:** Patient details of adverse reactions associated with EPSOLAY.

Case id	PT	Drug indication	Drugname	Administration route	Dose_form	Reporter	Adverse event start date – medication start date (days)
21172059	Application site erythema, application site pain, application site swelling, application site hypersensitivity	Not specified	EPSOLAY	Topical	Cream	Consumer	
21172060	Dry skin, erythema, pruritus	Rosacea	EPSOLAY	Topical	Cream	Consumer	0
21172061	Hypersensitivity, pruritus, skin exfoliation, swelling of eyelid, skin swelling, skin burning sensation	Not specified	EPSOLAY	Topical	Cream	Consumer	0
21191020	Dry skin, skin irritation	Product used for unknown indication	EPSOLAY	Topical	Cream	Physician	
21274488	Application site pruritus, dry skin, skin exfoliation, application site burn	Rosacea	EPSOLAY	Topical		Consumer	35
21568592	Drug ineffective	Product used for unknown indication	EPSOLAY	Topical	Cream	Consumer	
21568596	Erythema, pruritus, skin warm	Product used for unknown indication	EPSOLAY	Topical	Cream	Physician	0
21568598	Erythema, skin irritation, skin burning sensation	Product used for unknown indication	EPSOLAY	Topical	Cream	Physician	
21573627	Drug ineffective	Not specified	EPSOLAY	Unknown	Cream	Physician	
21969375	Dry skin, skin irritation, off‐label use, skin burning sensation, intentional underdose	Product used for unknown indication	EPSOLAY	Topical	Cream	Physician	
21969378	Dry skin, skin irritation, off‐label use, intentional underdose	Product used for unknown indication	EPSOLAY	Topical	Cream	Physician	
21969379	Acne, Intentional product use issue	Rosacea	EPSOLAY	Topical		Consumer	2
22305488	Eczema eyelids	Product used for unknown indication	EPSOLAY	Topical		Physician	
22305489	Dry skin, erythema, intentional underdose, intentional product misuse	Not specified	EPSOLAY	Topical		Consumer	
22305490	Acne, acne pustular, erythema, pruritus, skin hypertrophy, skin injury	Rosacea	EPSOLAY	Topical		Consumer	1
22305491	Application site erythema, hypersensitivity	Rosacea	EPSOLAY	Topical		Pharmacist	80
22743186	Underdose, expired product administered, inappropriate schedule of product administration	Rosacea	EPSOLAY	Topical		Consumer	
22743187	Product dispensing error	Rosacea	EPSOLAY	Topical		Consumer	
23111895	Application site erythema, skin burning sensation	Product used for unknown indication	EPSOLAY	Topical	Unknown	Consumer	
23111896	Erythema, pruritus	Rosacea	EPSOLAY	Topical	Unknown	Consumer	3
23138327	Application site rash, rash, application site vesicles	Rosacea	EPSOLAY	Not specified		Consumer	3
23488384	Drug ineffective for unapproved indication, intentional product use issue	Hidradenitis	EPSOLAY	Unknown	Gel	Pharmacist	
23500679	Erythema, pruritus, skin burning sensation, product use in unapproved indication	Rosacea	EPSOLAY	Topical	Unknown	Consumer	0
23821003	Erythema, eye swelling, lip dry, lip swelling, pain of skin, rash, skin exfoliation, skin swelling, skin burning sensation	Rosacea	EPSOLAY	Unknown	Unknown	Consumer	0
23821014	Drug ineffective for unapproved indication, intentional product use issue	Hidradenitis	EPSOLAY	Topical	Unknown	Physician	
23821027	Erythema	Product used for unknown indication	EPSOLAY	Topical	Unknown	Consumer	
23821028	Swelling face	Product used for unknown indication	EPSOLAY	Topical	Unknown	Consumer	

## 4 Discussion

BPO, as a therapeutic agent for skin treatment, exerts its antibacterial and anti‐inflammatory effects, thereby alleviating acne symptoms. It has been extensively utilized in the management of acne. Given the widespread application of BPO, evaluating its safety is of paramount importance. This assessment facilitates an understanding of BPO's stability under varying conditions, its toxicity, and potential impacts on human health.

Through the analysis of demographic characteristics in BPO adverse reaction reports, we observe that female patients dominate (74.18%), which may correlate with their greater tendency to report health issues or higher frequency of using beauty products, such as medications for acne treatment. However, this gender disparity underscores the need for heightened attention to safety among female patients using specific medications. The 18–44 age group accounts for the highest proportion of adverse reaction reports (37.45%), possibly linked to this demographic's heightened demand for beauty and skin care products. Notably, the majority of reports originate from consumers (61.90%), emphasizing the crucial role patients or consumer groups play in drug safety monitoring, albeit potentially influencing data completeness and accuracy. Furthermore, acne is the most prevalent indication (75.18%), indicating widespread market use of medications for this condition, which may also imply higher risks of adverse reactions. The majority of reports are categorized as severe (81.59%), underscoring the necessity for strengthened drug safety monitoring and risk assessment. Additionally, nearly half of the adverse reactions occur within the first day of medication use, highlighting the importance of early monitoring and intervention.

From the perspective of adverse reaction symptoms, the majority of cases are centered on dermatological symptoms commonly observed, such as edema, stinging sensations, and rashes. These symptoms may be attributed to the photosensitivity of BPO, which accelerates its decomposition upon exposure to sunlight, thereby enhancing its irritant and allergenic properties [10]. Consequently, special attention must be paid to sun protection during storage and usage to avoid direct sunlight exposure. It is imperative for physicians to adequately inform patients of these precautions [11].

Under specific environmental conditions, particularly when the storage temperature exceeds 25°C, BPO poses a risk of decomposing and generating benzene [12], a well‐established potent carcinogen [3]. This discovery has sparked profound concern and extensive discussions regarding the safety of BPO within the medical community and among the general public. Currently, the question of whether BPO is carcinogenic has emerged as a pivotal issue of utmost concern for patients and a critical research direction for scientists seeking to gain a clearer understanding through rigorous investigation. Our findings also identified multiple signals of adverse reactions associated with malignancy. However, because of the lack of information on duration, dosage, and other relevant factors, coupled with the fact that all reported cases were from consumers, we are unable to assess the reliability of our results. Currently, there have been some clinical studies indicating a potential association between BPO and certain types of cancer [13, 14]. It is widely acknowledged that the development of cancer may take an extended period of time; hence, short‐term clinical observations cannot fully alleviate concerns regarding this product. Consequently, further research with a longer time span and a larger sample size is imperative to substantiate its safety.

It is crucial to emphasize that BPO itself does not directly cause cancer, but rather, its decomposition product, benzene, possesses carcinogenic properties. Consequently, researchers have continually refined the production processes of BPO to minimize the release of benzene. This leads to the emergence of EPSOLAY, which incorporates a unique patented technology that encapsulates BPO within silicone‐based microcapsules. This design creates a barrier between the drug and the skin, allowing BPO to release slowly over time. This mechanism not only enhances the efficacy of the drug but also significantly diminishes adverse reactions. As a result of its excellent safety profile and effectiveness, EPSOLAY is well‐suited for long‐term patient use [15]. Currently, 27 patients in the FAERS database have reported adverse reactions to EPSOLAY, most of which are skin irritation. No tumor‐related adverse reactions have been reported so far, which may be due to the relatively late market launch of this drug. As a newly launched drug, continuous post‐marketing surveillance is still necessary to collect data on its safety and effectiveness in actual use, so as to evaluate the risks and benefits of the drug. Through this stage, we can further understand the long‐term effects and potential risks of the drug, as well as its performance in different patient populations [16, 17].

Given that the research relies on the FAERS database, its inherent limitations cannot be overlooked. Firstly, the primary data of the FAERS database originate from the United States, which may lead to insufficient consideration of drug response characteristics in non‐American populations, particularly among different racial skin types, thereby potentially overlooking the influence of potential racial differences. Secondly, as the drug is predominantly used in a home environment, and the majority of reporters are ordinary consumers rather than medical professionals, this may, to a certain extent, diminish the professionalism and objectivity of the evaluations, increasing the risk of subjective bias. Furthermore, the significant issue of missing data in the FAERS database poses a major obstacle to accurately analyzing the causes of ADRs, limiting the breadth and depth of in‐depth analysis.

Nonetheless, because of its vast data scale, the FAERS database continues to occupy a pivotal position in the global field of ADR monitoring, recognized as one of the most authoritative data resources in this domain. Its extensive coverage and abundant information provide invaluable reference points for the ongoing assessment and improvement of drug safety [18].

## 5 Conclusion

BPO, a commonly employed dermatological agent, holds significant importance in acne management. However, its potential adverse effects cannot be overlooked, particularly the carcinogenic risk associated with its decomposition product, benzene. By conducting a systematic analysis of adverse reaction reports within the FAERS database, this study uncovers the characteristics of BPO‐related adverse reactions across diverse populations, usage scenarios, and symptomatic manifestations. Furthermore, corresponding preventive measures and future research directions are proposed. Simultaneously, it addresses the limitations of current studies, including geographical biases in data sources and the non‐expertise of patient‐reported cases. Consequently, it is suggested that future investigations incorporate considerations such as racial disparities, environmental factors, and drug administration conditions to provide a more comprehensive assessment of BPO's safety profile.

## Ethics Statement

The authors have nothing to report.

## Consent

The authors have nothing to report.

## Conflicts of Interest

The authors declare no conflicts of interest.

## Data Availability

The data that support the findings of this study are available from the corresponding author upon reasonable request.
